# Can organized leisure-time activities buffer the negative outcomes of unstructured activities for adolescents’ health?

**DOI:** 10.1007/s00038-018-1125-3

**Published:** 2018-06-02

**Authors:** Petr Badura, Andrea Madarasova Geckova, Dagmar Sigmundova, Erik Sigmund, Jitse P. van Dijk, Sijmen A. Reijneveld

**Affiliations:** 1 Institute of Active Lifestyle, Faculty of Physical Culture, Palacký University, Tr. Miru 117, 771 11 Olomouc, Czech Republic; 2 Department of Community and Occupational Medicine, University Medical Center Groningen, University of Groningen, Groningen, The Netherlands; 3 Olomouc University for Society and Health Institute, Palacký University, Olomouc, Czech Republic; 4 Department of Health Psychology, Faculty of Medicine, Safarik University, Kosice, Slovakia; 5 Graduate School Kosice Institute for Society and Health, Safarik University, Kosice, Slovakia; 6 Department of Social Medicine and Public Health, Faculty of Medicine and Dentistry, Palacký University, Olomouc, Czech Republic

**Keywords:** Adolescence, Extracurricular activities, Unstructured leisure, Substance use, Sexual intercourse, School performance

## Abstract

**Objectives:**

We aimed to assess the associations of involvement in selected unstructured activities (UA) with health-risk behaviours and academic achievement and the degree to which the participation in organized leisure-time activities (OLTA) changes these associations.

**Methods:**

Using a sample of 6935 Czech adolescents aged 13 and 15 years, we investigated adolescents’ weekly involvement in hanging out, visiting shopping malls for fun and meeting friends after 8 p.m., OLTA and engagement in three health-risk behaviours and academic achievement.

**Results:**

Weekly involvement in the selected UA was associated with higher odds for regular smoking, being drunk, having early sexual intercourse and low academic achievement. Concurrent participation in OLTA did not buffer these negative outcomes, except for sexual experience. However, those highly engaged only in UA were more likely to participate in the health-risk behaviours and report worse academic achievement than those participating in any OLTA concurrently.

**Conclusions:**

The selected UA are strongly associated with an increased occurrence of adolescents’ health-risk behaviours and low academic achievement. Concurrent participation in OLTA does not buffer these negative outcomes significantly, but adolescents engaged only in UA consistently report the least favourable outcomes.

## Introduction

In western industrialized countries, leisure time comprises approximately half of adolescents’ waking hours, with a slightly higher amount of leisure time recorded in North America than in Europe (Larson and Verma 1999; Wight et al. 2009). Unlike school, household chores or personal care (e.g., sleep or hygiene), it offers room for a wide range of activities. Regardless of geographical location, it is therefore typified by more pronounced inter-individual differences in its content, as well as distinct associations with health and developmental indicators. Some sorts of activities (e.g., organized activities) can be considered as health-enhancing and supporting development (Larson 2000; Mahoney et al. 2006). Oppositely, some specific unstructured activities, such as frequent visits to shopping malls for fun or hanging out in public places on a regular basis, might pose a threat to adolescent health (Caldwell and Faulk 2013).

Engagement in unstructured activities (UA) is frequently associated with problematic outcomes, but not all UA can be labelled as risky (Bradley 2010; Sharp et al. 2015). UA that actually expose youth to health risks have the following characteristics: adult-unsupervised, lack of skill-building aims, taking place in public and especially having a strong socializing character (Mahoney et al. 2004; Osgood et al. 2005; Weerman et al. 2015). Data from both Europe and the U.S. support the conclusion that settings with such features offer adolescents space for engagement in risky behaviours (Augustyn and McGloin 2013; Lee and Vandell 2015) and are appealing to adolescents who are generally more vulnerable to these behaviours (Mahoney et al. 2004; Persson et al. 2007). Indeed, youth who spend a lot of time in such activities with little or no structure have been reported to have higher rates of substance use (Kiesner et al. 2010; Pulver et al. 2015), potentially risky sexual activity (Barnes et al. 2007) and to do worse in school, both in terms of grade point average and honours or recognitions earned (Nelson and Gastic 2009).

Organized leisure-time activities (OLTA) are in fact exactly opposite of such UA, as they are characterized by having a certain structure, a regular schedule, clearly defined goals and rules, focusing on skill-building and being adult-supervised (Larson 2000; Mahoney et al. 2006). In contrast to socializing UA, youth in OLTA experience higher levels of intrinsic motivation and challenge at the same time (Hansen et al. 2003). This promotes a development of initiative, identity formation, building of teamwork skills and social capital (Hansen et al. 2003) and links OLTA to healthy developmental outcomes (Farb and Matjasko 2012). Compared to those not participating in OLTA, participants in OLTA report better health (Badura et al. 2015; Leversen et al. 2012) or school performance (Badura et al. 2016; Fredricks 2012) and, oppositely, a lower occurrence of health-risk behaviours (Badura et al. 2017; Takakura 2015), as observed in studies from the U.S., Europe and Japan.

As is apparent from the findings described in the two preceding paragraphs, the developmental outcomes of OLTA (i.e., highly structured) and UA are contradictory. However, OLTA and UA obviously are not mutually exclusive categories of leisure time. A noteworthy group of U.S. adolescents participate in OLTA and, at the same time, are involved in a range of UA (Bartko and Eccles 2003; Sharp et al. 2015). However, little is known if (and how) outcomes for those concurrently involved in risky UA and OLTA are distinct from those involved only in UA. An understanding is needed of how the adolescents cope with combining these two sorts of leisure-time activities with ‘conflicting’ characteristics and outcomes regarding health-risk behaviours and school performance.

First, our study aimed to assess the associations between three ‘risky’ UA and indicators of healthy youth development (health-risk behaviours and academic achievement). Given the gender, age, as well as socioeconomic differences both in the leisure time content (Badura et al. 2015; Sharp et al. 2015) and health-risk behaviours (Inchley et al. 2016), we checked if these mentioned confounding factors modified the associations. Last, we also investigated the degree to which participation in OLTA changed these associations and how adolescents differed regarding the above indicators according to their involvement in unstructured and organized leisure-time activities.

## Methods

### Sample and procedure

Our data come from the Czech Health Behaviour in School-aged Children (HBSC) study, which was conducted between April and June 2014. The surveyed schools were randomly selected from the database of Ministry of Education, Youth and Sports of the Czech Republic. Out of the 244 schools approached after stratification by region and ratio of primary and secondary schools, 243 gave consent to conduct the survey. At each of the participating schools, one class from the 5th, 7th and 9th grades (corresponding to age categories 11, 13, and 15 years) was then picked up at random. The questionnaires were administered by trained research assistants during regular class time and in the absence of teachers to minimize potential response bias. Participation in the study was voluntary and anonymous, with no incentives offered to respondents. Prior to administration of the questionnaires, the respondents were notified about the possibility to opt out. The Ethics Committee of the Faculty of Physical Culture, Palacky University, Olomouc, approved the study design (No. 57/2014).

There were 16,298 pupils registered in the surveyed classes, and 14,539 of them completed the questionnaires. Thirty pupils refused to take part in the survey, and 1729 pupils were not present at school during the survey, with the most common reason being an illness. Then, we selected only 13- and 15-year-old adolescents, because the questions on UA were not asked to those aged 11 years. Finally, we excluded respondents who were classified as age outliers (e.g., a 15-year-old completing the questionnaire for 7th graders/13-year-olds) or failed to provide data on gender, OLTA or UA. Those missing one or two responses out of the three UA items were only included in the analyses if we could unambiguously classify them as highly engaged in UA, i.e., one of their valid responses was *daily* or two of their valid responses were *weekly or more often*.. The final sample comprised 6935 adolescents (49.1% boys).

### Measures

We investigated involvement in three various peer-oriented UA. The respondents were asked how often they (a) *met their friends after 8 o’clock*, (b) *visited shopping malls for fun or distraction*, (c) *hung out with their friends in their neighbourhood, park, at playgrounds,* etc. We then categorized them into those doing these activities *weekly or more often* versus those doing them *less frequently*. We assessed these three activities separately and also derived a composite variable of being involved in *any of the unstructured activities on a daily basis or in at least two such activities at least weekly* as an indicator of high engagement in UA.

Regarding OLTA, the respondents indicated whether they participated in the following six types of activities: *team sports*, *individual sports*, *art schools*, *youth organizations*, *leisure centres or after*-*school clubs*, and *church meeting/singing*. Those involved in at least one OLTA were categorized as *participants*, while the rest as *non*-*participants*. Last, we split the adolescents into four categories based on their leisure-time activities: (1) involved *neither in OLTA nor UA*, (2) involved *only in OLTA*, (3) involved *both in OLTA and UA*, and (4) involved *only in UA*.

We used four dependent variables in our analyses from the pool of the HBSC mandatory questions (Currie et al. 2014) and dichotomized them according to the most recent HBSC international report (Inchley et al. 2016). Academic achievement was measured using the question: *In your opinion, what does your class teacher think about your performance compared to your classmates?* The responses were dichotomized as above-average achievement (*very good/good*) versus the remainder (*average/below average*).

Current smoking was assessed by the question: *How often do you smoke tobacco at present?* Four response categories were dichotomized as *every day* or *at least once a week* versus *less than once a week* or *I do not smoke*.

Drunkenness was assessed by the question: *In the last 30* *days, have you had so much alcohol that you were really drunk?* The respondents were split into those who indicated being drunk *at least once in the last 30* *days* versus those *not being drunk in the last 30* *days*.

Last, we investigated lifetime experience with early sexual intercourse. This was done using the question *Have you ever had sexual intercourse (sometimes this is called “making love”, “having sex”,* etc*.)?*, with dichotomous response option *yes* versus *no*. The question was asked only to 15-year-olds.

Socioeconomic status of adolescents’ families served as a control variable in our analyses. It was assessed using the Family Affluence Scale (FAS) developed for the purposes of the HBSC study (Currie et al. 2014). The responses on six items (*car ownership*, *holidays abroad*, *having one’s own bedroom*, *number of computers in the household*, *number of bathrooms*, and *dishwasher ownership*) were summed up. We then transformed the sum into a fractional rank score (0–1) (Elgar et al. 2017), with a higher value indicative of a higher level of affluence.

### Statistical analyses

First, we described the sample, its involvement in UA and OLTA, its self-reported academic achievement and engagement in health-risk behaviours. The statistical significance of gender- and age differences was examined by Chi-square tests.

Second, we assessed the associations of specific UA (both the separate activities and the composite variable) with academic achievement and health-risk behaviours using binary logistic regression. This was done in five steps—a crude model (Model 1), adjusted for age and gender (Model 2) and further adjusted for socioeconomic status, as indicated by FAS (Model 3). For the composite variable (at least on UA daily or at least two UA weekly), we also tested the interaction with the association of gender (Model 4) and of age category (Model 5).

Next, we tested the ‘buffering effect’ of OLTA on the associations of UA (participation in at least two of them weekly) with academic achievement and health-risk behaviours. The OLTA variable was added to the model, and the ‘buffering effect’ was assessed by the interaction between OLTA and high engagement in UA. To assess the stability of our results, we also ran the regression analyses using the pattern of OLTA participation, as previously derived by cluster analysis (Badura et al. 2015), which is indicative of the breadth of such participation. The outputs were very similar to those reported in the paper, so for sake of brevity we used the simple dichotomous OLTA variable (at least one OLTA vs. none) in the end.

Lastly, we ran the logistic regression with four categories for the combination of OLTA and UA involvement as independent variables (with those involved only in OLTA and UA concurrently as a reference category) to assess the differences between adolescents involved both in OLTA and UA and the rest of the sample, especially those involved only in UA.

Multilevel analyses of the risk behaviours and school-related outcomes on the Czech 2013/2014 HBSC sample did not indicate the data to cluster by school (Badura et al. 2016, 2017). For this reason, we used ordinary single-level regression models to assess the associations in the present study.

## Results

### Involvement in unstructured and organized leisure-time activities

Out of the three investigated UA, hanging out with friends was the most prevalent one (Table 1). Approximately, half of the respondents indicated doing it several times a week. Around a quarter of them visited shopping malls for fun regularly, and 16% met their friends after 8 o’clock in the evening, with the latter activity being significantly more common among 15-year-olds than among younger adolescents. Slightly over one-third of the respondents reported being involved in any of these UA on a daily basis or two or more UA at least weekly. Regarding OLTA, almost 80% of respondents took part in one or more such activity, with slightly higher rates in boys than in girls, and in 13-year-olds than in 15-year-olds.

**Table 1:** Description of the study population: rates of respondents’ involvement in unstructured and organized activities, health-risk behaviours and self-reported academic achievement by gender and age category; health behaviour in school-aged children study (HBSC), Czech Republic, 2013–2014

	Gender		Age		Total	Missing values
	Boy (*n* = 3408)	Girl (*n* = 3527)	13 years (*n* = 3415)	15 years (*n* = 3520)	(*n* = 6935)	
*Unstructured activities*						
Hanging out (several times a week)	1548 (45.4%)	1884 (53.4%)*	1719 (50.3%)	1713 (48.7%)	3432 (49.5%)	2
Visiting shopping malls for fun (several times a week)	818 (24.0%)	1120 (31.8%)*	1027 (30.1%)*	911 (25.9%)	1938 (27.9%)	1
Meeting after 8 p.m. (at least weekly)	626 (18.5%)*	499 (14.2%)	363 (10.7%)	762 (21.8%)*	1125 (16.3%)	36
High overall UA engagement (at least one UA daily or two at least weekly)	1044 (30.6%)	1336 (37.9%)*	1155 (33.8%)	1225 (34.8%)	2380 (34.3%)	0
*Organized activities*						
Organized activities (at least one)	2732 (80.2%)*	2719 (77.1%)	2857 (83.7%)*	2594 (73.7%)	5451 (78.6%)	0
*Dependent variables*						
Current smoking (weekly)	299 (8.8%)	378 (10.8%)*	149 (4.4%)	528 (15.1%)*	677 (9.8%)	36
Drunkenness in the last 30 days (once or more)	415 (12.5%)	400 (11.6%)	180 (5.4%)	635 (18.5%)*	815 (12.0%)	153
Sexual intercourse^a^ (yes)	359 (21.2%)	423 (24.3%)*	*N/A*	782 (22.8%)	782 (22.8%)	86
Academic achievement (average or worse)	1603 (47.2%)*	1453 (41.5%)	1511 (44.5%)	1545 (44.1%)	3056 (44.3%)	42

*UA* unstructured activities; *N/A* not available; % represents relative rate of valid responses

*Statistically significant (*p* < 0.05) difference in relative rates by gender or age per variable—based on *χ*^2^ tests

^a^The item was present only in the questionnaire version for 15-year-olds

### Unstructured activities, health-risk behaviours and academic achievement

Table 2 presents odds ratios (OR) and 95% confidence intervals (95% CI) for the associations of the three selected UA with self-reported health-risk behaviours and academic achievement. Involvement in any of these three activities was significantly associated with higher odds for regular smoking, being drunk recently, having sexual intercourse, and low academic achievement. ORs for the univariable model (Model 1—not shown) and the models adjusted for the gender and age category (Model 2) and additionally FAS (Model 3) hardly differed. Meeting friends after 8 o’clock in the evening showed the strongest associations out of all the dependent variables.

**Table 2:** Odds ratios (OR) and 95% confidence intervals (CI) for low self-reported academic achievement, substance use and experience with sexual intercourse for adolescents engaged in unstructured leisure-time activities versus those not involved in these activities; health behaviour in school-aged children study (HBSC), Czech Republic, 2013–2014

	Current smoking (at least weekly)	Drunkenness last 30 days (once or more)	Sexual intercourse^a^ (yes)	Academic achievement (average or worse)
	OR (95% CI)	OR (95% CI)	OR (95% CI)	OR (95% CI)
*MODEL 2 adjusted for gender and age*				
Hanging out	3.31*** (2.76–3.98)	2.57*** (2.19–3.01)	2.33*** (1.97–2.75)	1.13* (1.03–1.25)
Visiting shopping malls for fun	2.33*** (1.97–2.76)	2.13*** (1.82–2.50)	2.30*** (1.93–2.73)	1.32*** (1.18–1.46)
Meeting friends after 8 p.m.	4.28*** (3.59–5.10)	4.22*** (3.58–4.97)	4.67*** (3.90–5.59)	1.47*** (1.29–1.67)
High UA engagement	4.75*** (3.98–5.65)	3.50*** (3.00–4.09)	3.66*** (3.10–4.33)	1.42*** (1.29–1.57)
*MODEL 3 adjusted for gender, age and FAS*				
Hanging out	3.30*** (2.75–3.96)	2.57*** (2.19–3.02)	2.35*** (1.99–2.77)	1.13* (1.02–1.24)
Visiting shopping malls for fun	2.35*** (1.99–2.79)	2.13*** (1.82–2.49)	2.29*** (1.93–2.72)	1.33*** (1.19–1.48)
Meeting friends after 8 p.m.	4.36*** (3.65–5.20)	4.22*** (3.58–4.97)	4.66*** (3.89–5.58)	1.49*** (1.31–1.70)
High UA engagement	4.75*** (4.00–5.66)	3.50*** (3.00–4.09)	3.68*** (3.12–4.35)	1.42*** (1.29–1.57)
*MODEL 4 interaction with gender, adjusted for age and FAS*				
Main effect—gender (boy vs. girl)	0.79 (0.60–1.04)	1.09 (0.86–1.36)	0.91 (0.72–1.16)	1.36*** (1.20–1.53)
Main effect—high UA engagement	4.31*** (2.84–4.44)	3.23*** (2.59–4.03)	3.68*** (2.92–4.63)	1.49*** (1.29–1.71)
Interaction—boy* High UA engagement	1.24 (0.87–1.75)	1.17 (0.86–1.59)	1.00 (0.72–1.40)	0.90 (0.74–1.11)
*MODEL 5 interaction with age, adjusted for gender and FAS*				
Main effect—age (15 vs. 13 years)	2.95*** (2.17–4.01)	3.71*** (2.84–4.84)	N/A	0.97 (0.86–1.09)
Main effect—high UA engagement	3.27*** (2.33–4.60)	2.99*** (2.20–4.07)	N/A	1.41*** (1.22–1.63)
Interaction—age 15* high UA engagement	1.64* (1.11–2.44)	1.23 (0.86–1.76)	N/A	1.02 (0.83–1.24)

*FAS* family affluence scale; *UA* unstructured activities; *N/A* not available

*p < 0.05; **p < 0.01; ***p < 0.001

^a^The question on sexual intercourse was asked only to 15-year-olds

None of the interaction effects of gender on the associations of high engagement in UA was statistically significant. Regarding interaction effects of age category, we observed only 15-year-olds who reported excessive engagement in UA to have higher odds of current smoking than 13-year-olds (Table 2; Model 5). The interactions with the other dependent variables were not statistically significant.

### ‘Buffering effect’ of organized activities on the negative outcomes of unstructured activities

Next, we assessed the potential ‘buffering effect’ of OLTA on the negative outcomes related to high engagement in UA (Table 3). We found an interaction effect of OLTA participation with involvement in UA regarding sexual intercourse (OR = 0.64; 95% CI = 0.44–0.93). This indicates that those engaged highly in UA were less likely to have a sexual experience when involved in OLTA, too, compared with those not involved in OLTA. None of the other interactions was statistically significant.

**Table 3:** Odds ratios (OR) and 95% confidence intervals (CI) for, substance use, experience with sexual intercourse and low self-reported academic achievement, including the interaction between involvement in unstructured and organized activities; health behaviour in school-aged children study (HBSC), Czech Republic, 2013–/2014

	Current smoking (at least weekly)	Drunkenness last 30 days (once or more)	Sexual intercourse^a^ (yes)	Academic achievement (average or worse)
	OR (95% CI)	OR (95% CI)	OR (95% CI)	OR (95% CI)
*Main effect*				
High UA involvement (at least one UA daily or two at least UA weekly)	5.35*** (3.87–7.40)	4.14*** (3.02–5.68)	5.16*** (3.73–7.14)	1.44** (1.15–1.81)
OLTA (at least one)	0.74* (0.55–1.00)	1.00 (0.77–1.31)	1.01 (0.77–1.32)	0.55*** (0.47–0.63)
*Interaction effect*				
≥ 1 OLTA* high UA involvement	0.87 (0.59–1.27)	0.81 (0.56–1.16)	0.64* (0.44–0.93)	1.01 (0.78–1.30)

The model above was adjusted for gender, age and FAS

*FAS* family affluence scale; *OLTA* organized leisure-time activities; *UA* unstructured activities

*p < 0.05; **p < 0.01; ***p < 0.001

^a^The question on sexual intercourse was asked only to 15-year-olds

Lastly, we also investigated the effects per combination of UA and OLTA, with adolescents involved in both OLTA and UA as a reference category, as they were of special interest to the present study (Table 4). Compared with adolescents highly engaged only in UA, those involved both in UA and OLTA had lower odds regarding all four dependent variables assessed; however, the OR regarding recent drunkenness was not statistically significant. Those involved in UA and OLTA concurrently were, nonetheless, more likely to smoke regularly and have experience with sexual intercourse than adolescents involved only in OLTA or uninvolved in any of the leisure-time activities—organized or unstructured—investigated in the study. On the other hand, and unlike adolescents only in OLTA, the uninvolved group had higher odds of doing worse at school than those involved both in OLTA and UA.

**Table 4:** Odds ratios (OR) and 95% confidence intervals (CI) for above-average self-reported academic achievement, substance use and experience with sexual intercourse for various combinations of leisure-time activities; health behaviour in school-aged children study (HBSC), Czech Republic, 2013–2014

	Current smoking (at least weekly)	Drunkenness last 30 days (once or more)	Sexual intercourse^a^ (yes)	Academic achievement (average or worse)
	OR (95% CI)	OR (95% CI)	OR (95% CI)	OR (95% CI)
Both OLTA and UA (*n* = 1905)	Ref	Ref	Ref	Ref
Only OLTA (*n* = 3546)	0.22*** (0.18–0.27)	0.30*** (0.25–0.36)	0.30*** (0.25–0.37)	0.69*** (0.61–0.77)
Neither OLTA nor UA (*n* = 1009)	0.29*** (0.22–0.39)	0.30*** (0.23–0.39)	0.30*** (0.23–0.39)	1.26** (1.07–1.47)
Only UA (*n* = 475)	1.56*** (1.22–1.99)	1.24 (0.97–1.59)	1.55** (1.19–2.03)	1.81*** (1.47–2.23)

The model above was adjusted for gender, age and FAS

*FAS* family affluence scale; *OLTA* organized leisure-time activities; *UA* unstructured activities

*p < 0.05; **p < 0.01; ***p < 0.001

^a^The question on sexual intercourse was asked only to 15-year-olds

## Discussion

The present study showed that participation in peer-oriented UA, such as regular hanging out, visiting shopping malls for fun and meeting with friends in the evening, was associated with more frequent smoking, getting drunk, having early sexual intercourse and performing worse in school. This was, in general, independent of respondents’ gender, age and socioeconomic status. Concurrent participation in OLTA did not buffer these negative outcomes, except for experience with sexual intercourse. However, adolescents involved only in UA indicated less favourable outcomes on regarding smoking, sexual intercourse and academic achievement than those involved concurrently in UA and OLTA.

Adolescents engaged in the selected UA at least weekly were more prone to substance use, sexual intercourse and low academic achievement compared with their unengaged peers. Our findings are in accordance with a significant body of literature that reported these sorts of activities to be predictive of antisocial and norm-breaking behaviours (Augustyn and McGloin 2013; Haynie and Osgood 2005; Hoeben and Weerman 2016) or linked to worse school performance (Bae and Wickrama 2015; Nelson and Gastic 2009) and increased rates of substance use (Lee and Vandell 2015; Spilkova 2015). UA appear to attract adolescents who generally incline towards health-risk and delinquent behaviours (Mahoney et al. 2004; Persson et al. 2007), and exposure to such peers is one of the frequently discussed reasons for the negative outcomes of UA (Haynie and Osgood 2005; Hoeben and Weerman 2016; Svensson and Oberwittler 2010). It is thus possible that risky behaviours and an inclination to UA involvement simply form interrelated features of an underlying personality trait.

However, there is also a complementary explanation for the association of high engagement in UA with health-risk behaviours. It seems that risk-taking in UA is driven mainly by situational motivation and, thus, the root cause lies inherently in the nature of these activities. They provide youth with opportunities for health-risk behaviours (Haynie and Osgood 2005; Hoeben and Weerman 2016; Persson et al. 2007; Siennick and Osgood 2012). This may also explain the fact that adjustment for gender, age and socioeconomic status hardly affected the associations observed. High engagement in unsupervised peer-oriented UA, therefore, appears to be risky across social strata for both adolescent boys and girls.

The ‘buffering effect’ of concurrent OLTA participation on negative outcomes of engagement in UA was not statistically significant, except for sexual experience. This is somewhat surprising, given the relatively strong evidence linking OLTA to healthy development (Farb and Matjasko 2012). However, it is in line with some authors who warned against exaggeration of the assumed ‘positive’ effects of participation in OLTA (Fredricks and Eccles 2006; Larson 2000), which could actually be weaker than previously suggested. Moreover, this finding advocates for studying more general patterns of leisure-time use, including a wider array of structured and unstructured leisure-time activities of adolescents when investigating their developmental outcomes (Nelson and Gastic 2009; Sharp et al. 2015).

However, those involved only in UA were more likely to engage in substance use, have sexual intercourse and have worse academic achievement than those involved in both OLTA and UA. Those with only UA and no OLTA might more often feel bored during leisure time or lack of meaningful leisure opportunities. This has been shown to be associated with increased rates of substance use (Weybright et al. 2015) and sexual activity (Miller et al. 2014). On the other hand, those that are concurrently involved in OLTA, implying being a member of a certain social group, might feel less need to stabilize their position through health-risk behaviours (Viau et al. 2015) and might also have less time and fewer opportunities to engage in such behaviours. Nonetheless, it is apparently not enough to participate in OLTA in order to avoid health-risk behaviours, but one should also avoid major UA involvement.

### Strengths and limitations

The main strengths of our study are its large nationally representative sample and use of the well-established HBSC methodology. However, our study has also some limitations. First, we used self-report questionnaires, which can be sensitive to bias (e.g., due to social desirability or recall bias). We limited this risk by administering the questionnaire anonymously in the absence of teachers. Moreover, most of the measures we used were well validated (Currie et al. 2014). Second, no conclusions regarding causality of the observed associations can be drawn due to the cross-sectional design of the study. Third, our dichotomous measure of OLTA participation can be considered somewhat crude. However, repeating the analyses also with a pattern of OLTA participation yielded similar results as those with dichotomized OLTA. Nonetheless, we still missed information regarding frequency, engagement in, or duration of OLTA participation, which could have provided deeper insight into the topic.

### Implications

Weekly involvement in UA, including hanging out, visiting shopping malls for fun and meeting friends after 8 p.m., was associated with significantly low academic achievement, regular smoking, drunkenness and experience with sexual intercourse. If causal, increase in youth-appealing leisure opportunities could lead to prevention of health-risk behaviours through reduction of UA involvement, because time is a finite resource. This was recently observed by Motamedi et al. (2016), particularly in girls. Next, parental awareness and control of their children’s leisure-time activities would perhaps act similarly, as concluded by previous research (Barnes et al. 2007; Kiesner et al. 2010).

Our study provides some hints that participation in OLTA may reduce the occurrence of health-risk behaviours, even though the ‘buffering effect’ of organized activities on negative outcomes of UA was not significant. More evidence is needed using more detailed measures of OLTA participation, because the measure we used was only dichotomous. Future research should, therefore, focus on particular dimensions of OLTA participation, such as intensity of participation, engagement or quality of program, with more detailed data on the participation probably providing more cues for understanding and intervening.

### Conclusions

Involvement in peer-oriented unstructured activities is strongly associated with an increased risk of smoking, getting drunk, experience with sexual intercourse and worse academic achievement in adolescence. Except for sexual experience, concurrent participation in organized activities did not significantly buffer these negative outcomes, but adolescents involved only in unstructured activities were the most at-risk group. They were more likely to smoke, experience sexual intercourse and do worse at school than those involved in organized and unstructured activities concurrently.
